# Sex dimorphism in inflammatory response to obesity in childhood

**DOI:** 10.1038/s41366-021-00753-1

**Published:** 2021-02-01

**Authors:** Estefania Simoes, Joanna Correia-Lima, Leonardo Sardas, Felipe Storti, Thais Zélia dos Santos Otani, Daniel Augusto Correa Vasques, Victor Henrique Oyamada Otani, Pamela Bertolazzi, Cristiane Kochi, Marilia Seelaender, Ricardo Riyoiti Uchida

**Affiliations:** 1 Cancer Metabolism Research Group, University of São Paulo, São Paulo, Brazil; 2 Mental Health Department, Santa Casa de Sao Paulo School of Medical Sciences, São Paulo, Brazil; 3 Physiology Department, Santa Casa de Sao Paulo School of Medical Sciences, São Paulo, Brazil; 4 Faculdade de Medicina, University of São Paulo, São Paulo, Brazil; 5 LIM 26, Hospital das Clínicas of the University of São Paulo, São Paulo, Brazil

**Keywords:** Risk factors, Paediatrics, Pathology, Body mass index

## Abstract

**Background:**

Childhood overweight and obesity are a global concern, with prevalence rising dramatically over the last decades. The condition is caused by an increase in energy intake and reduction of physical activity, leading to excessive fat accumulation, followed by systemic chronic inflammation and altered function of immune cell responses. This study aimed at providing new insights regarding sex-specificity on the inflammatory response to obesity in the young patient.

**Design:**

Forty-three Brazilian obese adolescents (Female = 22 and Male=21, BMI (body mass index) Z-score average = 2.78 ± 0.51) and forty-nine eutrophic adolescents (Female = 24 and Male = 25, BMI Z-score average = −0.35 ± 0.88) were enrolled in the study. Anthropometrical analyses and blood cell counts were carried out. Using Luminex®xMAP™ technology, circulating serum cytokines, chemokines, and inflammatory biomarkers were analyzed. Two-way ANOVA test, Tukey’s test, and Spearman’s correlation coefficient were employed, with a significance threshold set at *p* < 0.05.

**Results:**

We identified increased levels of serum amyloid A (SAA), platelets, and leukocytes solely in male obese patients. We found a noteworthy sex-dependent pattern in regard to inflammatory response: obese boys showed higher TNFβ, IL15, and IL2 and lower IL10 and IL13, while obese girls showed increased TNFα, CCL3, CCL4, and IP10 content in the circulation. BMI Z-score was significantly linearly correlated with neutrophils, leukocytes, platelets, SAA, TNFα, CCL3, CCL4, IP10, and IL13 levels within the entire cohort (non-sex-dependent).

**Conclusions:**

Our data support a complex relationship between adiposity, blood cell count, and circulating inflammatory cytokine content. High SAA levels suggest that this factor may play a critical role in local and systemic inflammation. In the eutrophic group, females presented a lower status of inflammation, as compared to males. Both obese boys and girls showed an increased inflammatory response in relation to eutrophic counterparts. Taken together, results point out to clear sex dimorphism in the inflammatory profile of obese adolescents.

## Introduction

Overweight and obesity are considered a worldwide epidemic, having nearly triplicated over the last decades in both industrialized and developing countries [1]. According to the World Health Organization (WHO), 39% of adults, women, and men aged 18 years and older were overweight in 2016. Of these, over 13% were obese, while at least 2.8 million adults will die each year as a result of this medical condition [2]. More of a concern, the prevalence of childhood overweight and obesity has risen dramatically, overall more than 124 million children and adolescents aged 5–19 are overweight or obese [3]. Also, a recent meta-analysis demonstrated that the presence of obesity in childhood was related to five-fold augmented risk of developing adult obesity, as compared with the risk of eutrophic children [4].

Obesity is a common condition caused by an increase in energy intake and a reduction of physical activity, leading to excessive fat accumulation [5]. Moreover, socio-cultural and environmental changes like sleep restriction or food consumption, as well as endocrine alterations, genetic predisposition, and epigenetic factors contribute to obesity development [6, 7]. Raised BMI is a significant risk factor for cardiovascular diseases (CVDs), dyslipidemia, type-2 diabetes (T2D), hypertension, cancer, and others [8]. Furthermore, a study showed that higher BMI during childhood (7–13 years of age) is associated with an increased risk of CVD in adulthood [9].

Obesity pathophysiology comprises systemic chronic inflammation and altered immune function [10, 11]. White adipose tissue has been characterized as a major source of inflammatory mediators, including secretion of cytokines, adipokines, free fatty acids, and regulation of acute-phase proteins [12], including tumor necrosis factor-α (TNF-α), interleukin (IL)−1, IL-10, IL-6, and chemoattractant proteins, all of which regulate adipogenesis and energy expenditure [13]. In juvenile obesity, inflammation is associated with early atherosclerotic vessel injury [14]. Furthermore, recent studies demonstrate that obesity is also associated with functional alterations of macrophages, natural killer (NK), and lymphocyte count and function [1, 15]. There remain, nevertheless, major gaps in data regarding alteration in white blood cell (WBC) count in childhood and juvenile obesity [16, 17].

To the best of our knowledge, molecular determinants triggering these disrupted responses, including childhood population characteristics and sex-specific aspects, are still poorly known. Previous studies show sex-related differences in the inflammatory status in CVD [18, 19]. Naturally, physiological alterations in inflammatory markers in males relative to females are expected, given the distribution of adipose tissue, hormonal response, and body composition as a whole. From this point of view, the present study was designed to elucidate the differences of inflammatory markers and blood cell count in a cohort of obese adolescents, with a special focus on sex dimorphism. Taking into account sexual differences, our data suggest new insights regarding the importance of sex effect on inflammatory response and blood cell count in childhood obesity.

## Materials and methods

### Participants

All subjects were 12–17 years of age and recruited between April 2014 and July 2016, at the children obesity outpatient clinic of the Santa Casa de Misericordia Hospital in São Paulo (SCMHSP). Participants were further divided: 49 Brazilian adolescents (Female: *n* = 24 and Male: *n* = 25, BMI Z-score average = −0.35 ± 0.88) were enrolled in the eutrophic group (control) with a BMI z-score above or equal to one; and forty-three obese adolescents (Female: *n* = 22 and Male: *n* = 21, BMI Z-score average = 2.78 ± 0.51) participated in the obesity group with a BMI z-score greater or equal to two. None of the subjects had current or previous clinical, neurological and psychiatric illnesses, nor the history of cranioencephalic trauma, seizure, neither previous neurosurgery. The study was approved by the SCMHSP Ethics Committee (CAAE: 24552413.2.0000.5479) and all parents and subjects signed an informed consent according to the Declaration of Helsinki.

### Anthropometrics and blood examination

Bodyweight and height were measured to calculate BMI using the Quetelet index (kg/m^2^). As a widely screening tool for obesity in children and adolescents, BMI z-scores were calculated by measuring relative weight adjusted for child age and sex following body mass index-for-age growth charts [20, 21]. Approximately 6 mL of blood was collected, between 7 a.m. and 7:30 a.m., by a trained health professional from the SCMHSP, into BD Vacutainer® Tubes containing or not anticoagulant (EDTA) after overnight fasting. The blood sample was centrifuged to obtain plasma and serum, respectively, for posterior analysis. Complete blood count (erythrocytes, lymphocytes, neutrophils, basophils, leucocytes, eosinophils, monocytes, and platelets) was performed with an automated blood cell counter (XE-2100; Sysmex, Roche). The neutrophil-to-lymphocyte ratio was calculated using the equation NLR = Neutrophil/Lymphocyte. Santa Casa de Misericordia Hospital performed the hemoglobin content measurement (Sysmex Roche XC2100), following standard hematologic test procedures. The serum concentration of fasting blood glucose (Diagnostic Roche Cat: #04404483190) and SAA (Merck Millipore—human cardiovascular disease magnetic bead panel Cat: #HCVD2MAG-67K) were quantified using commercial kits.

### Inflammatory cytokine analysis

Eighteen cytokines and inflammatory biomarkers were analyzed from the serum of patients, employing Luminex®xMAP™ technology. Multiplex assays were performed according to the manufacture’s protocol: human cytokine/chemokine magnetic bead panel Cat. #HCYTMAG-60K-PX30. The detection of target antigens bound to the microspheres was performed with a mixture of biotinylated capture antibodies after incubation for 1 h followed by incubation with streptavidin labeled with phycoerythrin for 30 min. Protein quantification was preceded by the use of the equipment software (xPONENT^®^ 4.2) and the obtained data were analyzed in the MILLIPLEX^®^ Analyst 5.1 Software. Data were expressed in picograms per milliliter (pg/ml).

### Data analysis

Data are expressed as mean ± SD (standard deviation). Data graphics were created using raw data. Gaussian distribution test was employed for all continuous variables (Kolmogorov–Smirnov test). Non-parametric data were transformed into normal distribution by applying a mathematical function (log or square root) to make the statistical analyses feasible with a two-way ANOVA test with multiple comparisons possible. Post hoc pairwise comparisons Tukey’s test was performed when appropriate. Spearman’s correlation coefficient and linear regression were used to assess the simple relationship between the variables. The significance threshold was set at *p* < 0.05. All statistical tests were performed with Graphpad Prism 7.0.

## Results

The general characteristics of 92 subjects are shown in Table 1. The obese and eutrophic cohorts were of similar age and height (*p* > 0.05). As expected, obese males and females had increased weight, BMI, and BMI Z-score (*p* < 0.0001). Hemoglobin levels were different depending on the factor sex, females presented lower levels than males in both, eutrophic and obese group (*p* < 0.0001). No differences in glucose levels or NLR (neutrophil-to-lymphocyte ratio) were found among groups. SAA, an important acute-phase reactant protein and inflammatory adipokine, was highly expressed in obese male patients compared to eutrophic males (*p* < 0.0001).

**Table 1:** Anthropometrical and biochemical data of eutrophic and obese cohorts.

	Eutrophic		Obese		Two-way ANOVA		
	Female (*n* = 24)	Male (*n* = 25)	Female (*n* = 22)	Male (*n* = 21)	Sex	Obes	SxO
Age (years)	14.46 ± 2.13	14.20 ± 1.94	13.82 ± 2.02	13.67 ± 1.96	0.627	0.166	0.899
Height (m)	1.58 ± 0.08	1.64 ± 0.13	1.62 ± 0.06	1.66 ± 0.11	0.510	0.167	0.700
Weight (Kg)	47.25 ± 9.38	51.23 ± 12.44	83.65 ± 15.44	86.76 ± 18.24	0.235	**<0.0001**	0.884
BMI (kg/m^2^)	18.80 ± 2.33	18.83 ± 2.41	31.99 ± 5.17	31.32 ± 4.06	0.679	**<0.0001**	0.649
BMI Z-score	−0.38 ± 0.85	−0.27 ± 0.94	2.69 ± 0.51	2.83 ± 0.53	0.443	**<0.0001**	0.954
Hemoglobin (g/dL)	13.66 ± 1.16	14.57 ± 0.98	13.12 ± 0.86	15.1 ± 1.37	**<0.0001**	0.992	**0.045**
Glucose (mg/dL)	88 ± 6.67	93.14 ± 6.79	90.41 ± 12.90	90.5 ± 5.60	0.210	0.956	0.226
SAA (pg/ml)	369.1 ± 864.9	126.9 ± 331.0	545.6 ± 742.3	678.1 ± 1138	0.069	**0.0001**	0.318
NLR ratio	1.81 ± 0.82	1.17 ± 0.51	1.64 ± 0.76	1.78 ± 0.63	0.143	0.195	0.07

Data presented as mean ± SD. Significance between the groups was tested using two-way ANOVA.

*N* number of patients, *NLR* neutrophil-to-lymphocyte ratio, *Obes* Obesity factor, *SxO* sex and obesity factors interaction.

Significant *p*-values in bold.

### Blood cell count alterations associated to sex or obesity

**Fig. 1:**
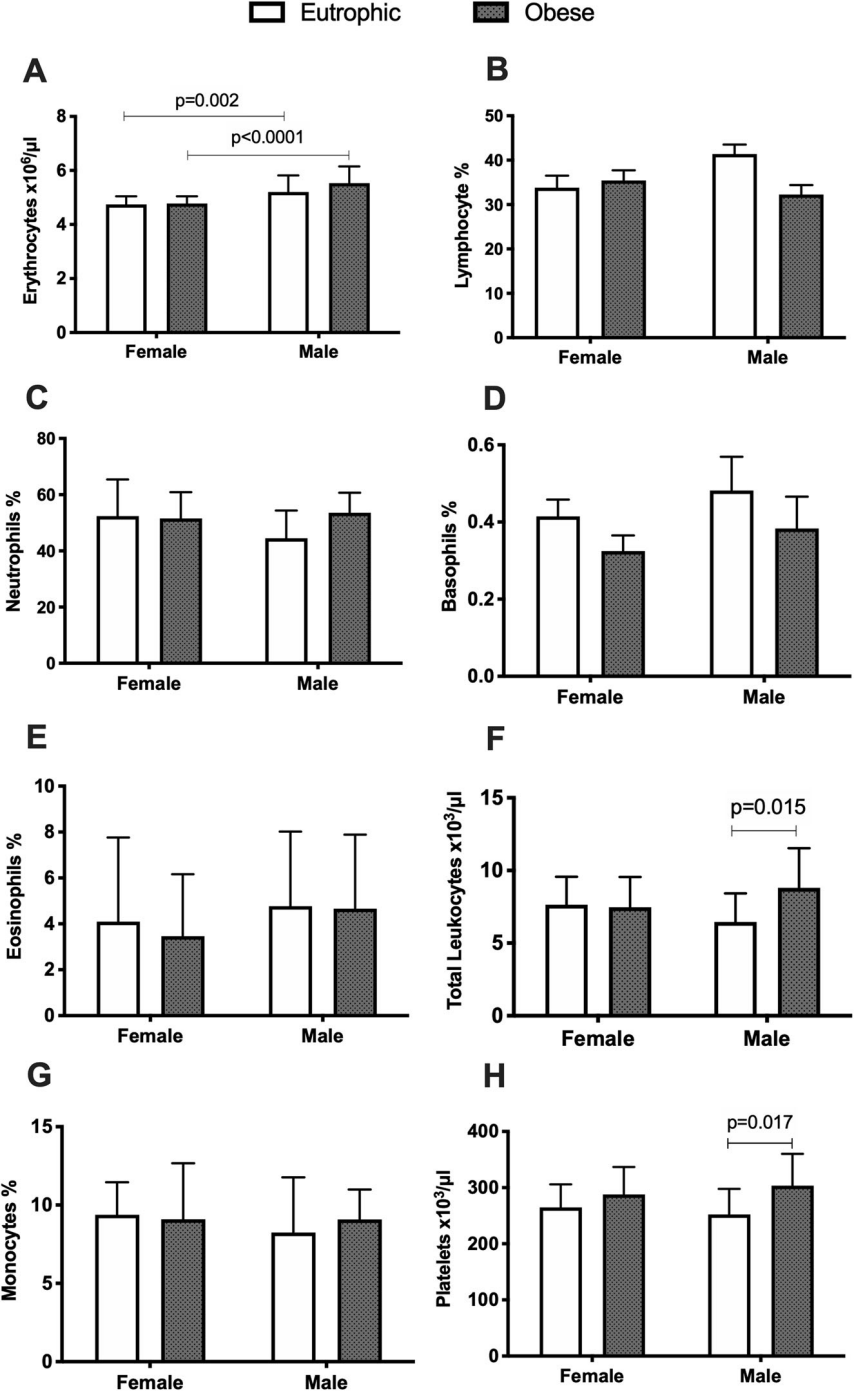
Complete blood cell count of enrolled patients. Eutrophic female, *n* **=** 23–24; eutrophic male, *n* **=** 25; obese female, *n* **=** 22; obese male, *n* **=** 21. Data presented as mean ± SD. Significance between the groups was tested using Post hoc pairwise comparisons Tukey’s test. Significant difference *p*-value < 0.05.

The complete blood count of the subjects is illustrated in Fig. 1. The total count of peripheral erythrocytes (*p* < 0.0001) was significantly different depending on the sex factor, with males presenting higher levels than females, in eutrophic and obese groups (Fig. 1a). In addition, higher count of total lymphocytes (*p* = 0.039) was found for eutrophic males compared to eutrophic females (Fig. 1b). Total leukocyte (*p* = 0.015, Fig. 1f) and platelet (*p* = 0.017, Fig. 1h) counts were significantly increased in obese males, relative to eutrophic males (obesity factor-dependent). Leukocyte number and platelets in obese or eutrophic females did not present differences, demonstrating that alterations of blood cell parameters in childhood obesity also present sex-dependence (occurring solely in boys). No differences were found in neutrophil, basophil, eosinophil, or monocyte counts among groups (Fig. 1c, d, e, g).

**Fig. 2:**
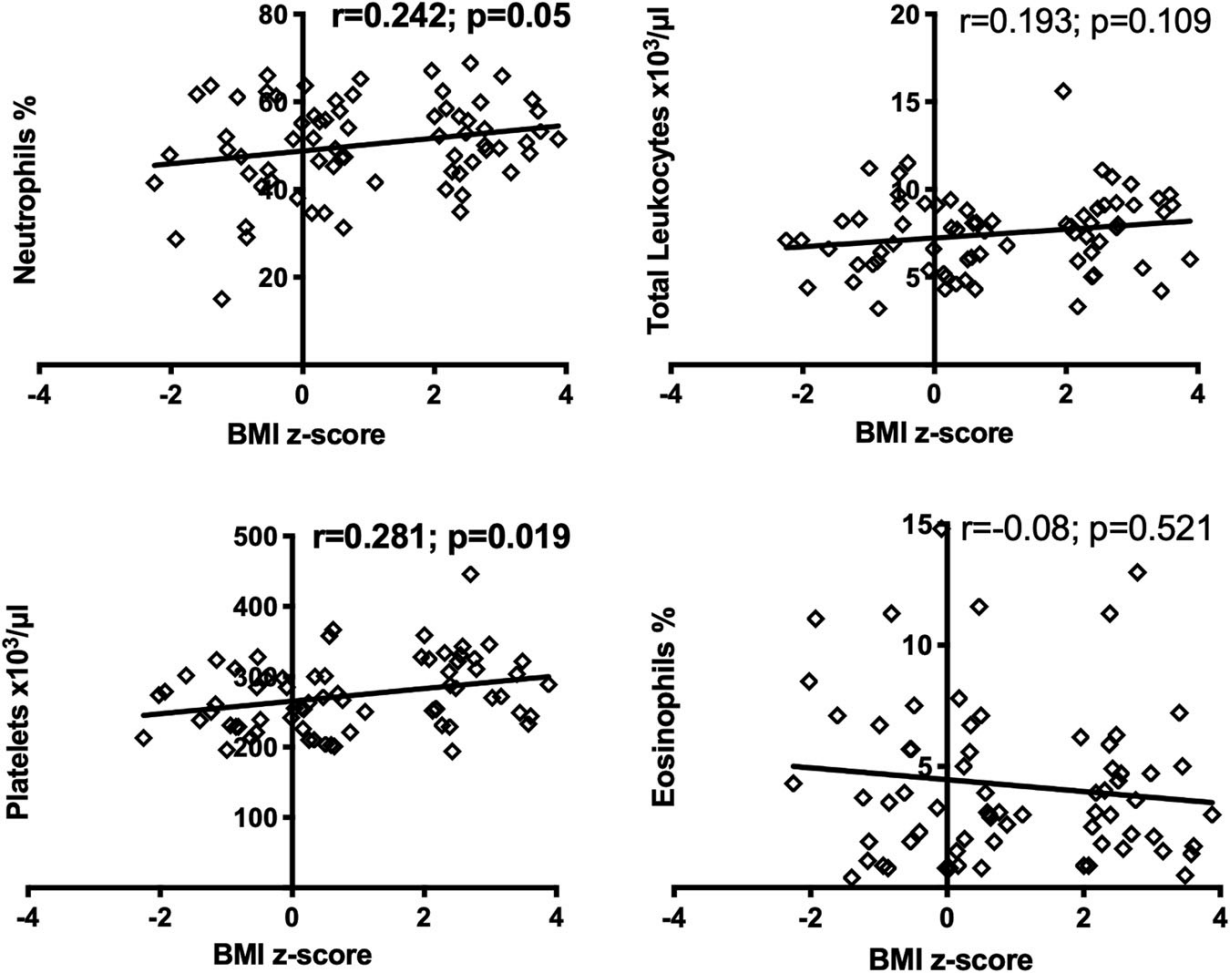
Spearman correlation of blood cells and BMI Z-score index in the entire cohort. Significant correlation *p* < 0.05 in bold. BMI body mass index.

Spearman analysis was performed to measure correlations between BMI Z-score and blood cell counts considering the entire cohort. The BMI Z-score positively correlated with Neutrophils (*r* = 0.242; *p* = 0.05), and platelets (*r* = 0.281; *p* = 0.019) in the circulation. No correlation was found between BMI Z-score and leukocytes (*r* = 0.109; *p* = 0.193), or eosinophils (*r* = −0.08; *p* = 0.521) (see correlation data in Fig. 2).

### Sex-specific pattern of inflammatory markers in childhood obesity

Circulating inflammatory markers in childhood obesity were analyzed (Table 2).

**Table 2:** Sex-specific pattern of inflammatory markers in childhood obesity.

	Eutrophic		Obese		Two-way ANOVA		
pg/ml	Female (*n* = 24)	Male (*n* = 25)	Female (*n* = 22)	Male (*n* = 21)	Sex	Obes	SxO
TNF-α	5.53 ± 2.75	9.99 ± 4.46	9.30 ± 3.84	9.47 ± 3.16	**0.003**	**0.035**	**0.005**
TNF-β	3.43 ± 7.25	7.93 ± 8.29	1.69 ± 2.33	1.96 ± 3.47	**0.025**	**0.011**	0.293
VEGF	37.58 ± 28.21	190.9 ± 298.0	736.2 ± 650.3	124.4 ± 317.2	0.059	0.740	0.153
IL-1α	3.66 ± 9.01	37.04 ± 36.8	1.98 ± 4.52	7.83 ± 18.13	0.052	0.515	0.874
IL-1RA	5.30 ± 4.56	13.65 ± 11.86	9.42 ± 9.64	9.1 ± 11.53	0.070	0.965	**0.040**
IL-2	1.12 ± 0.35	2.41 ± 1.36	1.26 ± 0.64	3.65 ± 11.42	**0.006**	0.268	0.085
IL-4	55.54 ± 61.65	55.91 ± 71.99	87.51 ± 100.5	91.21 ± 110.0	0.780	0.134	0.694
IL-6	1.42 ± 0.58	2.31 ± 1.58	1.78 ± 0.85	1.74 ± 0.74	0.065	0.903	**0.045**
IL-8	9.29 ± 6.74	12.35 ± 12.62	11.3 ± 13.15	10.09 ± 9.21	0.610	0.736	0.566
IL-10	1.89 ± 2.54	3.83 ± 5.89	1.64 ± 1.28	2.50 ± 4.54	**0.018**	0.165	0.106
IL-12P40	3.58 8.32	21.44 ± 23.32	3.16 ± 6.12	18.94 ± 76.57	0.052	0.538	0.997
IL-13	1.84 ± 3.24	4.34 ± 4.55	0.97 ± 0.83	0.80 ± 0.25	**0.020**	**0.003**	0.121
IL-15	1.47 ± 0.54	3.30 ± 2.04	2.57 ± 4.43	3.51 ± 9.23	**0.005**	0.164	**0.008**
IL-17A	1.95 ± 1.53	6.22 ± 15.47	3.56 ± 6.59	5.05 ± 12.50	0.087	0.684	0.277
IP-10	183.7 ± 121.1	326 ± 321.9	290.2 ± 217.5	317.9 ± 217.4	0.079	**0.039**	0.285
CCL2	462.1 ± 175.7	652.3 ± 358.3	539.2 ± 379.5	555.1 ± 312.4	0.100	0.745	0.240
CCL3	3.23 ± 2.82	152.5 ± 513.1	121 ± 301.8	6.37 ± 5.22	0.173	**0.021**	**0.0001**
CCL4	23.42 ± 12.79	37.5 ± 15.79	42.35 ± 17.56	40.84 ± 23.80	0.054	**0.003**	**0.015**

Data presented as mean ± SD. Significance between the groups was tested using two-way ANOVA.

*N* number of patients, *Obes* Obesity factor, *SxO* sex and obesity factors interaction.

Significant *p*-values in bold.

Comparing eutrophic females and males post hoc analyses (control group), we found that males showed significantly increased concentration of circulating inflammatory factors, such as IL-6 (*p* = 0.005), IL-1RA (*p* = 0.005), TNF-α (*p* < 0.0001), TNF-β (*p* = 0.016), IL15 (*p* = 0.0001), IL-2 (*p* = 0.001), IL-10 (*p* = 0.004), IL-13 (*p* = 0.005), IP-10 (*p* = 0.039), CCL3 (*p* = 0.0002), CCL4 (*p* = 0.001), relative to eutrophic females. Nevertheless, when analyzing obese males and females, we observed different patterns of alterations in the inflammatory markers. Systemic inflammation in obese boys was characterized by higher expression of TNF-β (*p* = 0.011), IL-15 (*p* = 0.004), and IL-2 (*p* = 0.046) and significantly lower concentrations of anti-inflammatory cytokines such as IL-10 (*p* = 0.035) and IL-13 (*p* = 0.002), compared to eutrophic males. On the other hand, obese females showed significantly higher expression of TNF-α (*p* = 0.0007) and increased expression of chemokines, such as CCL3 (*p* < 0.0001), CCL4 (*p* = 0.0002), and IP-10 (*p* = 0.027). No significant differences were found among groups for serum IL-12P40, IL-1α, IL-4, IL-8, VEGF, or IL-17A.

**Fig. 3:**
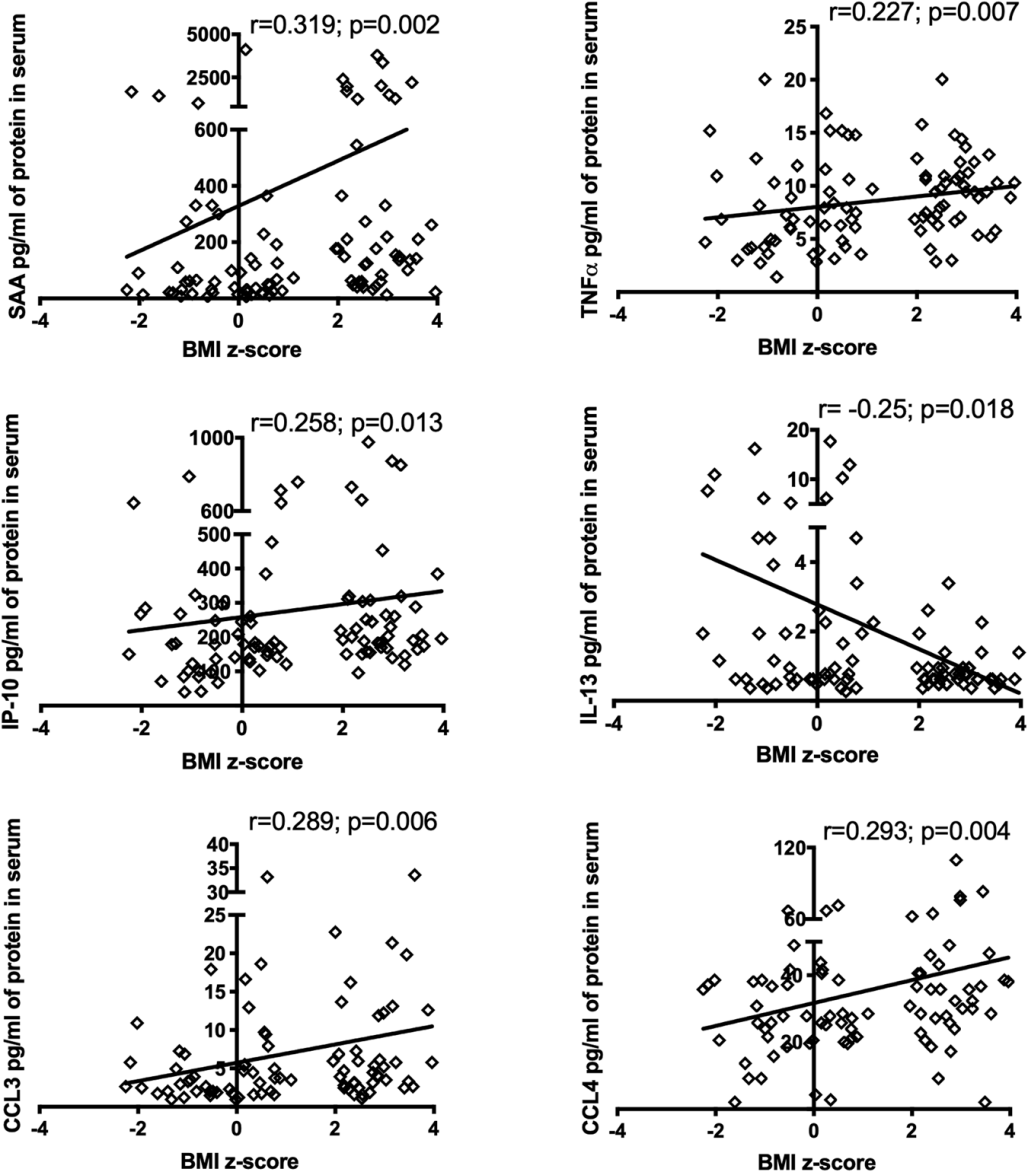
Spearman correlation of BMI Z-score and inflammatory markers. Spearman correlation significance *p* < 0.05. BMI body mass index, SAA serum amyloid A, TNF-α tumor necrosis factor A, IL interleukin, CCL chemoattract.

Spearman analysis was carried out to measure the correlations between BMI Z-score and inflammatory factors, taking into account the entire cohort. BMI Z-score positively correlated with SAA (*r* = 0.319; *p* = 0.002), TNF-α (*r* = 0.227; *p* = 0.007), CCL3 (*r* = 0.289; *p* = 0.006), CCL4 (*r* = 0.293; *p* = 0.004), and IP-10 (*r* = 0.258; *p* = 0.013) (see Fig. 3). However, BMI was negatively and linearly related to anti-inflammatory cytokines IL-13 (*r* = −0.25; *p* = 0.018) (see Fig. 3).

## Discussion

In the present study, we evaluated a Brazilian eutrophic and obese adolescent cohort, in an attempt to elucidate possible sexual dimorphism in childhood obesity responses. More specifically, we addressed blood cell count and inflammatory factors in the circulation. Anthropometrical measurements as age and height were homogeneous between eutrophic and obese patients. Obese males and females showed a higher BMI Z-score compared to the eutrophic group, as expected. Biochemical data analysis showed significant differences in hemoglobin depending on the sex factor. In contrast with several studies in obese adults, we did not find alterations in glucose serum content in the cohort. We nevertheless, demonstrated that SAA, an acute-phase protein synthesized in response to infection, inflammation, injury, and stress was upregulated in obese males compared to the other groups. SAA was described as a protein predominantly expressed and released by the liver [22] but, recently several studies demonstrated that during the non-acute-phase reaction, the human adipose tissue is a major site of SAA expression [23, 24]. Recent studies have shown that obese patients exhibit significantly increased circulating SAA concentration, positively correlated to the size of adipocytes [25, 26].

Furthermore, we investigated in the Brazilian juvenile cohort the correlation of obesity, and blood cell count with circulating inflammatory markers and the differences in girls and boys. Our data show that the number of platelets and leukocytes was solely higher in obese males, while obese females did not show significant differences, in contrast with Charles et al., who reported a higher count of platelets in women, but not in men [27]. Nevertheless, other studies with Iranian subjects showed a significant correlation between waist circumference and the number of platelets in both male and female subjects, and the platelet counts were significantly increased in obese women [28]. Moreover, the count of erythrocytes and lymphocytes was significantly different in eutrophic and obese patients, according to sex factor, comparing females and males. Correlation analyses revealed that a greater BMI Z-score was significantly associated with increased cell counts, including neutrophils and platelets. Recently, studies suggest that the count of WBCs in obesity can be employed as an indicator of clinical inflammation and as a marker in predicting the direct correlation of obesity and the risk of diabetes [29].

As mentioned before, obesity pathophysiology is characterized by systemic chronic inflammation, commonly mediated by cytokines [10, 11]. It is well known, that obesity is associated with increased circulating levels of TNF-α, IL-6, and reactive oxygen species. In accordance with previous studies that reported sex differences in the inflammatory responses [19, 30, 31], our data show an important sex-dependent difference comparing eutrophic males and females, with significantly higher IL-6, IL-1RA, TNF-α, TNF-β, IL15, IL-2, IL-10, IL-13, IP-10, CCL3, and CCL4 in control males. In addition, we identified a marked difference in cytokine patterns between obese males and females. Is well know that during the transition into puberty and then, adulthood, endocrine events are very relevant and the differences imposed by sex may elicit heterogeneous input in the mechanisms of obesity [32]. Nevertheless, to our knowledge, inflammation and anti-inflammatory markers have not been comprehensively examined in this context. In our study, we found that circulating inflammatory markers profile in obese males was characterized by increased expression of TNF-β, IL-15, and IL-2. IL-15 and IL-2 have several similar functions and are highly expressed in obesity [33]. Moreover, obese males showed significantly lower concentrations of anti-inflammatory cytokines as IL-10 and IL-13 compared to eutrophic males, clearly perpetuating a state of chronic inflammation. Low expression of anti-inflammatory cytokines, such as IL-10 has been associated with obesity and metabolic syndrome [34, 35]. From this point of view, previous studies demonstrated that circulating levels of IL-13 are reduced in diabetic patients that exhibit increased insulin resistance [36] and IL-13 overexpression protects against high-fat-diet-induced obesity [37].

Cytokine-related inflammation in obese females, was associated with higher expression of TNF-α, in line with Ziccardi et al. previous results [38]. TNF-α is considered an adipocytokine positively correlated with adiposity [39–42]. In addition, our study demonstrated the augment of chemokines such as CCL3, CCL4, and IP-10 in obese females. CCL3 and CCL4 are machophage-derived inflammatory proteins belonging to the CC-motif cytokine family, that show increased expression in diabetic patients and obesity [43, 44]. Also, chemokine interferon (IFN)-gamma-inducible protein-10 (IP-10/CXCL10) has been reported to be highly expressed in morbidly obese patients [45] and Chang et al. revealed that IP-10 is an independent risk factor associated with progressive liver injury, insulin resistance, and incident diabetes, indicating this to be a potential biomarker for disease progression and subsequent diabetes and non-alcoholic fatty liver disease (NAFLD) [46]. Other cytokines including IL-12P40, IL-1α, IL-4, IL-8 did not present significant (*P* < 0.05) differences among our groups.

We performed univariate analysis to evaluate the correlation between BMI Z-score and inflammatory factors for the whole cohort, as shown in Fig. 3. BMI Z-score is positively correlated with SAA, indicating that a higher concentration of SAA could contribute to obesity-associated CVD risk. Furthermore, BMI Z-score was positively correlated with inflammatory markers, such as TNF-α, IP-10, CCL3, and CCL4 content. Also, it was negatively correlated with anti-inflammatory cytokines like IL-13.

In conclusion, our data point out to the existence of a complex relationship between adiposity, blood cell count, and circulating inflammatory factor content. We found a noteworthy sex-dependent pattern in regard to the inflammatory response. Eutrophic group comparisons demonstrated that females present lower inflammatory status, as compared to males. On the other hand, obese males and females both show markedly increased inflammatory response in relation to eutrophic counterparts, with a clear sex dimorphism in the inflammatory profile in these obese adolescents.

The present study demonstrated signs of persistent inflammation and sex dimorphism in the inflammatory profile in juvenile obesity. We believe that prevention could reduce adult obesity-related complications and inflammation assessment may be a valuable strategy for early monitoring of adiposity. Further studies should address the effectiveness and feasibility of the use of the inflammatory profile in juvenile obesity as a start-point for interventions. Moreover, efforts to prevent and treat childhood and adolescent obesity should be encouraged.

Limitations of our study should be acknowledged for future follow-up studies: other methods for body composition analyses are more reliable than BMI or BMI Z-score, currently employed; the cohort was carried out with a juvenile Brazilian population sample and maybe not translatable to other populations.
